# Knowledge, Skills, and Support Needed by Teaching Personnel for Managing Challenging Situations with Pupils

**DOI:** 10.3390/ijerph16193646

**Published:** 2019-09-28

**Authors:** Pihla Markkanen, Minna Anttila, Maritta Välimäki

**Affiliations:** 1 Department of Nursing Science, University of Turku, 20014 Turku, Finland; minant@utu.fi (M.A.); mava@utu.fi (M.V.); 2 School of Nursing, Hong Kong Polytechnic University, Hong Kong, China

**Keywords:** comprehensive school, teaching personnel, pupil, challenging situation, mental health, focus group interview

## Abstract

It is not uncommon for teachers to face challenging behavioral issues in their classrooms, including disruptive, aggressive, or insulting behavior toward peers or adults. In this paper, we describe what knowledge, skills, and support is needed among teaching personnel to manage challenging situations with pupils. This study was carried out in one comprehensive school in Southwest Finland. Two focus group interviews were conducted with teaching personnel (schoolteachers and classroom assistants, *N* = 16). The participants also wrote short texts about challenging situations they had experienced. The qualitative data were analyzed with inductive content analysis. According to the results, the teaching personnel needed better knowledge about the factors affecting pupils’ behavior and about good practices to apply with pupils in challenging situations. Moreover, the personnel lacked the skills needed to anticipate and recognize pupils’ moods and signs of mental distress, and expressed the desire for support from mental health professionals. Teachers with adequate knowledge about the factors linked to behavioral issues are more capable of promoting environments conducive to positive interactions with their pupils, thereby limiting challenging situations. When developing education and support for teaching personnel, collaboration between education and mental health professionals is essential.

## 1. Introduction

Finland is internationally known for its highly ranked education system [1,2]. Its success has been attributed to the Finnish teacher training system, including the high level of education that teachers obtain, namely a master’s level university education, and teachers’ strong work motivation. These factors have enabled good performance by pupils in the Programme for International Student Assessment (PISA) [3], although some deterioration in the PISA results has been reported in recent years [4]. According to the United Nations Children’s Fund (UNICEF), Finland has one of the most equal education systems for children and adolescents as well as the second lowest level of inequality in well-being among children in the world [2,5].

Even though the value of a good education and welfare system has been acknowledged in global reports [2,5,6], economic deprivation has also been reported as being associated with the well-being of children. This was seen in the early 1990s in Finland, when society was facing an economic depression. For example, the municipalities that made the largest monetary cuts in school health services later ended up using more specialized psychiatric services for children and adolescents [7,8]. A longitudinal cohort study conducted in 1994 regarding children born in 1981 found that budget cuts in schools’ resources and teachers’ decreased work motivation were associated with an increased use of psychiatric services among pupils [7]. Thus, recent budget cuts in the Finnish public education system have raised concerns. For example, between 2010 and 2016, government expenditure on education as a percentage of the gross domestic product (GDP) declined from 6.6% to 6.1%, a lower expenditure than in the other Nordic countries, although still above the European Union average [9]. 

Meanwhile, behavioral problems among pupils, which affect the daily working lives of teachers, have become more common. Based on health-related statistics, the number of psychiatric and neurodevelopmental diagnoses among children and adolescents has increased in several high-income countries [10]. As the use of child and adolescent specialized services for psychiatric disorders has increased [11], the concern for how teachers cope with pupils with diverse needs has risen; since the aim is educational equity in schools, teachers must carefully manage pupils’ challenging behaviors to meet this goal [4,12,13,14,15]. There is still a gap between adolescents needing professional help and those who are getting treatment. For example, a prospective population-based study in Finland found that an estimated 7% of preadolescents had received professional help for behavioral or emotional problems [16]. However, according to a review study about the prevalence of mental health problems among children and adolescents, less than half of the adolescents with mental health problems get professional help when needed [17]. Adolescents with mental health problems may not receive needed help because of the lack of mental health services, their mental health needs are not recognized, or they do not want to the seek help for these issues. However, the increase in using specialized services in Finland may also be due to the changes in the help-seeking process, more people recognizing the problems, and increased resources for mental health services [11]. Not all children and adolescents with mental health issues need specialized services, but they should all be supported in primary care, for example, in the school setting. According to Gyllenberg et al. (2018), the number of suicides among adolescents in Finland was found to be low, that is, 16 suicides among the 1987 cohort and 12 among the 1997 cohort. Most of the adolescents who committed suicide had not used psychiatric services. Moreover, adolescents who need mental health services need to be more efficiently identified [11].

Dealing with the challenging behavior of pupils is one of the most worrying issues for teachers [18]. Pupils may behave in disruptive, aggressive, or defiant ways toward peers or adults [12,13,14,15], and they may disturb others in the class [12]. Teachers may feel incompetent in supporting pupils’ well-being [14,19] or dealing with misbehavior [12]. Teachers have reported that they have found pupils’ frequent off-task behavior, such as failing to complete work or putting one’s head down on their desk, to be very challenging. Pupils’ off-task behavior may also lead to other types of challenging behavior, for example, disruption or aggressive behavior [13]. Challenging behavior in school environments can be caused by attention deficit/hyperactivity disorder (ADHD) [20], conduct disorders or antisocial behavior [21], a pupil’s level of psychosocial stress, or problems in over-activity, inattention, and impulsivity [20,21]. Another challenge is bullying, as an average of 4% of pupils in the Organisation for Economic Co-operation and Development (OECD) countries have reported that they are hit or pushed by their peers at least a few times a month [22]. 

Managing pupils’ behavior at school is a challenge for teachers [23,24], yet teacher training often does not adequately prepare future teachers for managing the behavioral needs of diverse learners [25,26]. Teachers may not be capable of choosing the right ways of responding to pupils’ behavior, which may escalate the problem [27]. Teachers’ low self-efficacy in classroom management is associated with challenging behavior in the class as well as with the emotional exhaustion of teachers [28]. In Baker’s (2005) study, the likelihood of less experienced teachers’ taking action against bullying was associated with perceived efficacy but not with perceived seriousness [23]. Teachers may perceive that behavioral challenges are mainly caused by external factors, such as a pupil’s family situation, or that problems are controllable by the pupils. These types of perceptions may lead to a negative approach in dealing with pupils’ challenging behavior, and may result in the teacher resorting to punishments or refusing to use positive behavioral interventions [29]. Other reasons for children or adolescents displaying challenging behavior may be due to learning difficulties or disabilities [30] or cultural differences [31]. Sometimes teacher–pupil interaction patterns may be associated with a pupil’s behavior. Children who have a positive and supportive relationship with their teacher are less likely to have behavioral problems later [32]. On the other hand, it is not clear what kinds of pupils’ behaviors are considered challenging by teachers. In the study by Orsati and Causton-Theoharis (2013), challenging behavior is defined as social construct that depends on the contexts, in particular the rules for social environments in the classroom. Pupils that do not comply with the rules are often considered as challenging. When teachers have a positive relationship with pupils, they may be able to see past the rules and control and support pupils’ learning in the classroom [33]. An OECD survey (2017) found that bullying is more common in schools where pupils have negative relationships with their teachers [22]. 

Although these problems have led to the development of various strategies and interventions aimed at reducing pupils’ challenging behavior and promoting prosocial behavior [34,35,36], there is still room to better understand the knowledge and support needed among teaching staff [18,23,29,37,38]. Therefore, it is important to gain a more profound understanding of what types of support, knowledge, and skills teaching personnel need in school environments and what kinds of situations they have found challenging.

## 2. Materials and Methods 

The purpose of this study was to describe what knowledge, skills, and support are needed among teaching personnel for managing challenging situations with pupils. The research questions were as follows:

(1) What kinds of situations dealing with pupils have teaching personnel found challenging?
(2) What kinds of knowledge and skills related to the management of challenging situations at school do teaching personnel need?
(3) What kind of support related to the management of challenging situations at school do teaching personnel need?
(4) What kinds of suggestions related to current practices in schools have teaching personnel made?

### 2.1. Methodological Orientation

A descriptive qualitative study design was used to explore the perceptions of teaching personnel. The data were collected through focus group interviews and participants’ descriptive writings to elicit thoughts, perceptions, and new ideas in dealing with challenging situations with pupils at school, and to gain an understanding of the current situation for teaching personnel at school [39,40]. 

### 2.2. Setting

This study was conducted in one Finnish comprehensive school in Southwest Finland, which provides education for 578 pupils, of which 309 are in primary school (Grades 1–6) and 269 are in junior secondary school (Grades 7–9). Based on the Finnish Education Act (628/1998), compulsory education begins at the age of seven and is required by law [41]. Comprehensive school is government funded [39]. According to a PISA, Finnish schools are similar to each other, as there is little variance in students’ literacy scores between schools [1]. A family’s selection of a school is mainly based on the proximity to home rather than the school’s success or standing, that is, pupils usually attend the nearest school in their neighborhood [42]. As the aim of Finnish society is to support educational equity and inclusion, special needs education is mainly provided in mainstream schools. For example, children with special needs are provided with free auxiliary services, such as a classroom assistant [39]. 

### 2.3. Population and Sampling

The study population consisted of teachers, special education teachers, and classroom assistants. In Finland, class teachers in primary school have a master’s degree in education. In junior secondary school, subject teachers have a master’s degree in the subject they teach in addition to pedagogical studies [42]. Special education teachers are trained at universities, and their training concentrates on reading, language, mathematical, and behavioral issues [43]. Classroom assistants, who study for one year at a vocational institute, support pupils with special needs during the school day [44].

### 2.4. Recruitment 

Purposive sampling was used to recruit participants with experience and knowledge about the topic [45]. At the school, there were 55 teachers, 9 special education teachers, and 14 classroom assistants, and they all were informed about the study. First, an email was sent to the principal of the school describing the study. The principal informed the teaching personnel about the study and told them participation was optional. Second, the researcher (M.V.) visited the school to inform the staff about the purpose of the study and to discuss the topic. Out of 78 possible participants, 16 (20%) voluntarily agreed to participate in the focus group interviews. 

### 2.5. Description of the Participants

Altogether, eight teachers or special education teachers and eight classroom assistants participated. Participants were working with pupils of different ages, from the first grade to the ninth grade. Out of the 16 participants, one was male and the rest were females. Their ages varied from 23 to 59 years (mean 39.75, standard deviation (SD) 9.66). The length of their working experience varied from 1 to 20 years (mean 8.50, SD 7.43). 

### 2.6. Data Collection

The data were collected in April 2013. Two focus group interviews with 16 participants (9 and 7 participants in each group) were conducted. Teachers, special education teachers, and classroom assistants were present in both interviews, and they all discussed the questions together in order to promote a dialogue and new ideas among professionals who deal with challenging situations with pupils in their everyday work. The data collection included individual written texts and discussions from the focus group interviews.

After obtaining written informed consent, the interviewees were informed about the study, its voluntary nature, and their right to refuse or withdraw from the study at any point. The participants supplied their background information on a separate information sheet. Second, the participants were asked to describe in written format one challenging situation they had experienced at school. About 15 min were allowed for writing. The purpose of the written description was to stimulate discussion in the focus group. 

Third, four open-ended questions based on the research questions were addressed in the interviews: (1)“Please describe what kind of knowledge is needed in managing challenging situations with pupils at school”, (2) “Please describe what kinds of skills are needed in managing challenging situations with pupils at school”, (3) “Please describe what kind of support is needed in managing challenging situations with the pupils”, (4) “Do you have suggestions to improve the current situation at school?”.

Interviews were audio-recorded with permission of the participants in the presence of two interviewers—one interviewer took care of the recording and made notes, and the other focused on the content of the interview. The recorded interviews lasted altogether 1 h and 14 min (30 and 44 min each). All the participants wrote short descriptions about a challenging situation (or situations) they had experienced with pupils. The length of the texts varied from 29 words to 151 words.

### 2.7. Analysis

The data were analyzed using inductive content analysis, and only manifest content was analyzed. First, the audio-recorded data were transcribed verbatim and analyzed together with the participants’ descriptive writings [46]. The written data included a total of 27 pages (with a 2 cm wide margin on each side of the text, and a line spacing of 1.5).

Second, the transcribed data were read through several times to obtain a sense of the whole data [46]. Third, the unit of analysis was selected to be a phrase or a descriptive paragraph answering the research question [46], for example, describing a challenging situation. Fourth, the analysis units were abstracted and labelled with codes. Fifth, the codes were assigned to subcategories according to differences and similarities. Finally, the main categories were developed by grouping together the subcategories similar in meaning [46,47]. Examples of the data analysis process are presented in Table 1. 

### 2.8. Trustworthiness of the Study

The trustworthiness of this study is reflected in its credibility, conformability, dependability, and transferability [47,48]. Supporting credibility, all the participants had experiences of challenging situations with the pupils. They differed in professional background and age, which increases the possibility to gain information about the topic from a variety of aspects [46,47].

To ensure conformability, the analysis process was described carefully [47]. During the analysis, the written data were first coded and categorized, and then another researcher recoded the abstracted meaning units, subcategories, and main categories. All the subcategories and main categories were agreed upon, but there were some differences in coding, which were discussed and resolved [46].

Dependability refers to the stability of the data at different times and in different contexts [47]. In this study, the study context and the setting and decisions made during the process are reported. This allows the readers to follow the study process. The study was conducted at one school, and the results are not transferable, but readers can make their own decisions about applicability in other contexts [46].

### 2.9. Ethical Considerations

Permission to conduct the study (19.4.2013, §75) was received from the principal of the school. According to Finnish research regulations (Finlex 488/1999) and the regulations of the local research committees, no statement from the ethics committee was necessary [49,50]. The study did not involve patients, and all the participants were recruited on a voluntary basis and signed a consent form [51]. The participants were informed about their rights to withdraw from the study at any time without consequences. They were also able to contact researchers for further information. All personal information remained confidential and anonymous. Focus groups as a method reveals participants to each other, but the individually written descriptions were collected anonymously and without any personal information. The results are reported in such a way that the school and the participants are not recognizable to the readers. The data were accessible only to the researchers and stored in a secure place [45,51]. 

## 3. Results

### 3.1. Challenging Situations with Pupils

Challenging situations with pupils were found to be common for teaching staff. These situations occurred suddenly and were difficult to predict. According to the participants, challenging situations often erupted when the teachers intervened with a pupil’s behavior. Pupils were also found to have conflicts with their peers; the teachers reported physical fights and pupils annoying each other. The challenging behavior that was described was categorized as externalizing challenging behavior, socially withdrawn challenging behavior, and opposition.

#### 3.1.1. Externalizing Challenging Behavior

Externalizing challenging behavior was comprised of restless and impulsive behavior, running away from the situation, and physical and verbal aggression. Pupils’ unpredictable and impulsive behavior was reported as difficult to deal with. There were reports of pupils running out of the school during a conflict as well as verbal and physical aggression occurring between pupils and teaching personnel or between peers. One teacher’s written description of verbal and physical aggression is as follows: “The teacher intervenes, and the pupil hits and kicks everyone near him, bites, spits, and cannot be calmed down with talking or touching”.

#### 3.1.2. Socially Withdrawn Challenging Behavior

Socially withdrawn challenging behavior was described as passive aggression or as difficulties in contacting the pupil. The teaching personnel had many concerns about pupils behaving in passive aggressive ways, and those with whom the teaching personnel could not make contact were found to be unpredictable. Socially withdrawn pupils’ behavior was discussed in Focus Group 1: “I feel like I’m not able to connect, I can’t make eye contact, I can’t connect with speech; those kinds of pupils are the most unpredictable”.

#### 3.1.3. Opposing Challenging Behavior

Pupils were reported to sometimes directly oppose a teacher’s instructions, for example, by refusing to do their schoolwork or not sitting down in class. Opposition was also described as taking place in externalizing or socially withdrawn ways, as one participant described in writing:

“The pupil refuses to sit down in a specific place, there is a sharp exchange between the teacher and the pupil”.

### 3.2. Knowledge and Skills Needed for the Management of a Challenging Situation

The knowledge and skills needed for managing challenging situations were divided into two main categories—requirements for managing challenging situations and knowledge about issues related to those events. Some of the participants also reported that they did not know what kind of knowledge they needed. 

#### 3.2.1. Requirements Related to the Management of Challenging Situations

The knowledge requirements for managing challenging situations consisted of needing to know appropriate responses to pupils’ behavior, knowledge about early symptoms of aggression, and skills to recognize them and other signs of mental distress. Participants also needed guidance in how to handle the rest of the class during a conflict and were concerned how these incidents affected other pupils. The participants desired guidance in the right ways to interact with aggressive pupils and how to act in threatening situations. The ability to control one’s own emotions was considered important in dealing with challenging situations as were social skills for calming down an aggressive pupil. The participants discussed the need for such knowledge in Focus Group 2, for example, “How does one interact with an aggressive child? There wasn’t anything at all about that in our education”.

#### 3.2.2. Knowledge about Issues Affecting Pupils’ Behavior

The participants expressed the need for a better understanding of issues affecting challenging situations, such as pupils’ mental health problems, learning disabilities, and neuropsychiatric problems, as well as the skills needed to interact with pupils with different symptoms or diagnoses. This included general knowledge about these issues; teachers felt that basic knowledge about neuropsychiatric problems such as ADHD or the autism spectrum would increase their understanding and ability to support these pupils in their everyday environment. They desired more knowledge about mental health, for example, how to support a pupil’s learning and schooling if that pupil was depressed. Participants also felt that they did not always get all the important information about pupils’ special needs because of the lack of communication in the school. For example, a class teacher might know the best way to take their pupil’s special needs into account, and that knowledge could be shared with the subject teachers to further benefit the pupil. More knowledge and skills to recognize the symptoms of mental health problems and to evaluate pupils’ well-being and ability to do schoolwork were also needed. This especially concerned depressive or silently suffering pupils. The need for knowledge about pupils’ mental health was discussed in Focus Group 2: “… if you don’t know enough about what kind of child they are, how depression can manifest itself, or how in a million different ways it can manifest itself, that’s where my own knowledge runs out”.

### 3.3. Type of Support Needed for the Management of Challenging Situations and Suggestions for Improvement

The support needed for managing challenging situations fell into the following three categories—collegial support, multi-professional support, and work counseling. Various suggestions were offered by the participants on how to improve the current practices in the school setting.

#### 3.3.1. Collegial and Multi-Professional Support and Work Counseling

First, teachers described the collegial support to be good and readily available. Teachers described that the possibility to discuss with a colleague about challenging situations immediately after they happened supported their well-being at work. Moreover, discussions between colleagues helped when it came to making decisions about how to act in challenging situations with pupils. The collegial support was discussed in Focus Group 1: “Most often, problems can be solved in discussions with colleagues”.

Second, the support and counseling from the special education teachers regarding pupils’ special needs are essential for teachers. Teachers and classroom assistants often work together in pairs with the same class, and the mutual support was considered important. On the other hand, a lack of communication between teachers and classroom assistants was noticed. For example, when classroom assistants picked up pupils from a classroom, knowledge about any challenging situations that have just taken place could help them to anticipate any problems and support pupils. Classroom assistants expressed the feeling of being excluded from meetings where these issues were discussed. One participant (Focus Group 1) described multi-professional support as follows: “Yes, these incidents can be discussed straight away with the nearest working pair or with the special education teacher”. 

Third, the participants also needed professional work counseling so they could discuss challenging and emotionally stressful situations. Work counseling is professional counseling aimed at supporting one’s well-being at work. It enables reflective and confidential discussions with a professional counselor [52]. At the time of the study, the participating teaching personnel did not have access to work counseling. This topic was discussed in both focus groups. For example, one teacher (Focus Group 1) described collegial support as available, “but what is missing is undoubtedly work counseling”.

#### 3.3.2. Suggestions for Improvement

Regarding ways to improve the current practices in the school environment, the participants suggested that, first, more support from psychiatric professionals was necessary. They proposed that mental health nurses could work in schools to support children with behavioral or mental health problems and to counsel teaching personnel about their concerns. One participant (Focus Group 2) described the need for support from mental health professionals as follows: “The acute help, like the need to get someone to check out the situation in our classroom. At that point, one could get a mental health or other professional there to check it out with me and see where we are going”. 

Second, pedagogical groups for each grade level were requested, so that teaching personnel working with the same grade level could discuss their concerns with each other and enhance their collaboration. At the organizational level, participants desired more discussion about how they could conduct and improve their class management. Pedagogical groups were discussed in Focus Group 1: “… about pedagogical groups, they could really be useful, so those teachers would not be left alone”. 

## 4. Discussion

This study found that challenging situations are often related to pupils’ behavior, and that teaching personnel’s need for more knowledge and skills was related to managing pupils’ challenging behavior and anticipating challenging situations in order to promote positive interactions with pupils. According to the results, teaching personnel were receiving collegial and multi-professional support related to the management of challenging situations, but they thought that having access to mental health work counseling could also be helpful for discussing stressful situations. The participants made suggestions for improving the current practices at the school; for example, they hoped for collaboration with mental health professionals in the school setting and requested that there should be more discussion at the organizational level about the management of challenging situations. The participants suggested that they could start pedagogical groups themselves to enhance their collaboration and to improve the collegial and multi-professional support.

In this study, participants described situations where challenging situations were often related to pupils’ behavior. Likewise, according to earlier studies, challenging situations occur when teachers intervene in pupils’ behavior [12,14,15]. The study showed that teaching personnel need a better understanding of the issues affecting pupils’ behavior and better knowledge about managing challenging situations. This would include information about the right ways to act and the early symptoms of aggression and how to recognize them and other signs of mental distress. The need for teachers to have support and knowledge about how to manage challenging situations has also been recognized in earlier studies (e.g., Stormont et al. (2011) [38]). According to Baker’s study (2005), teachers’ perceived self-efficacy in managing challenging situations can predict their willingness and ability to manage those situations [23]. A teacher’s behavior and interaction with their pupils influence the pupils’ behavior [32], and a teacher’s ability to choose the right ways to act and intervene can prevent the escalation of problematic situations [27]. For example, an argument about seating may be avoided by asking the pupil where they want to sit. Therefore, it is important that teachers have enough support and the appropriate training in managing challenging behavior and in supporting the well-being of pupils. Receiving appropriate training should increase a teacher’s repertoire of strategies from which they can choose in challenging situations, and increase their understanding about how their own behavior affects and may trigger challenging behavior among their pupils [27,32].

The study found that the participants needed a better understanding of the factors affecting pupils’ behavior, such as mental health or neuropsychiatric problems. According to previous studies, when teachers have sufficient knowledge about ADHD [53] and the autism spectrum [54], they can better support the learning and the well-being of pupils with these special needs. Teachers also play an important role to play in recognizing pupils’ mental health needs and referring them to school health services. This need for teachers to be better informed about the mental health of their pupils has been highlighted in previous studies as well [55,56]. However, teachers should see their pupils as whole persons, instead of just diagnoses, and create a positive relationship with each pupil, since it promotes a good atmosphere and can help to prevent challenging behaviors [22,32]. The ability to see past the rules and control in the classroom promotes a good relationship between teacher and pupil, and the teacher may not so easily consider the pupil’s behavior as challenging. This prevents conflicts and promotes good atmosphere as well [33].

The need for improved communication among teaching personnel concerning pupils’ personal issues was also recognized. In this study, all the participants worked at the same school, and the need to improve their collaboration by enhancing communication arose from experiences in their everyday work. Learning from collaboration can make teaching personnel better equipped to anticipate these situations and promote a positive interaction with pupils. Positive relationships between teaching personnel and pupils promote a positive atmosphere in the school and help prevent bullying [22]. Moreover, low self-efficacy in classroom management is related to teachers’ emotional exhaustion [28], and teachers’ work-related stress can be reduced by learning effective ways to manage pupils’ challenging behavior and by improving teacher–pupil relationships [57]. 

According to the teachers’ perceptions, support from mental health professionals is needed in the school setting. Most of the children and adolescents who receive professional mental health care are treated in out-patient clinics, and they continue their schooling as usual. However, not all adolescents with mental health problems get professional help when needed, and teaching personnel regularly encounter pupils with mental health problems in their work [17,58]. Teaching personnel expressed concerns about how to identify those pupils who have mental health problems and how to evaluate the well-being of pupils and their ability to do the schoolwork. It has also been highlighted in previous studies that teaching personnel have an important role to play in recognizing the first signs of mental health problems among pupils and facilitating early intervention services [19,38,55,59]. Although teachers are not directly responsible for the care of pupils with mental health problems, they feel that they should support pupils’ well-being in addition to their learning [14]. Therefore, better knowledge about mental health issues would increase understanding about pupils’ behavior and might help the teaching personnel to support pupils’ mental well-being and to recognize those in need of professional help [55]. Moreover, the collaboration between teaching personnel and mental health professionals should be developed within schools.

### Limitations 

There were some limitations in this study. First, purposive sampling was used. The teaching personnel (*N* = 78) at the school were informed about the study, and 16 of them volunteered to participate. The sampling may have been biased toward those who were motivated and willing to discuss a sensitive topic with their colleagues [44]. It is possible that those teachers and classroom assistants who felt they were not coping with challenging situations with pupils were uncomfortable sharing their thoughts and experiences and did not volunteer. Whether this is the case remains unknown. 

Second, there was only one male participant and 15 females. Gender-based segregation in professional fields is common in Finland, compared to other countries [60], and in 2016, 77% of the teachers in basic education were females [61]. Therefore, it is not surprising that most of the participants were females, but the female teachers may have also been more willing to participate in the study than male teachers.

Third, all participants worked at the same school. Some of them might have felt it was difficult to talk about this topic with their colleagues from the same school, and this may have affected their readiness to participate and also the discussion [45]. However, it seemed to the researchers that the atmosphere in the focus group interviews was good, and participants could discuss the topic in a confidential manner.

Fourth, the purpose of the descriptive writings of one challenging situation was to stimulate discussion in the focus groups. While the time allowed for writing was about 15 min, the length and extent of the written descriptions varied. More data collection in written format could have opened up new thoughts about challenging situations at the school. On the other hand, not everyone was willing or able to write long essays. Therefore, a combination of writings and interviews was selected as a data collection format in order to ensure rich and as reliable data as possible. 

Fifth, the data were collected in 2013. The number of pupils with special needs has increased throughout mainland Finland since then. The intensified support arranged for comprehensive pupils was 6.5% in 2013 and 9% in 2016. The share of pupils in comprehensive schools receiving part-time special education did not change drastically in three years, namely, 20.3% in 2013 and 20.4% in 2016 [62]. The budget cuts in the Finnish public education system are still affecting the schools as they did in 2013 [9], but other changes in the school’s situation may have taken place.

In addition, the study was undertaken at only one school. Therefore, the results may not be generalizable to other schools, to different learning environments, or to other regions and countries.

Despite the limitations identified here, this study yielded important knowledge about what kinds of support and knowledge educational professionals need in their work. Understanding the teachers’ point of view is essential when developing vocational education and implementing evidence-based practices in schools. Teachers should have the ability to manage and anticipate challenging situations in the classroom and support pupils with special needs and challenging behavior. This promotes pupils’ well-being and their ability to learn and study at school.

## 5. Conclusions

Teaching personnel need to be more knowledgeable about mental health issues and have access to concrete support from mental health professionals in school settings. These issues are important to consider when developing effective support and vocational education for teaching personnel. Pupils should be supported in their everyday environments, and the ability of teaching personnel to deal with challenging situations promotes healthy school environments.

In practice, this study provides knowledge about the challenging situations in school environments. According to our results, teaching personnel find challenging situations difficult to predict, and these situations often occur when they intervene in pupils’ behavior. Teaching personnel need to better understand the issues affecting pupils’ behavior, such as neurodevelopmental issues and mental health. Teachers should know how to recognize the early symptoms of mental distress and aggression in order to be able to anticipate challenging situations. Training and vocational education including knowledge about the management and anticipation of challenging situations should be available for all professionals—class teachers, subject teachers, special education teachers, and classroom assistants—as they all deal with these situations in their work with pupils. This study encourages teaching personnel to develop collegial support in pedagogical groups and promotes communication between professionals. This may improve the handling of challenging situations and therefore support a more positive classroom atmosphere.

Further research should be carried out to find out the most effective ways to arrange support for teachers and increase their awareness about challenging situations at school. 

## Figures and Tables

**Table 1:** Examples of meaning units, codes, subcategories, and main categories.

Meaning Unit	Codes	Subcategories	Main Categories
A child got upset with another pupil and behaved aggressively, they were wrestling in the yard	Aggressive behavior, pupils were fighting	Externalizing challenging behavior	Challenging situations with pupils
How to interact with an aggressive child, there wasn’t anything about that in our education	Information about the right ways to act with an aggressive child	Requirements related to the management of challenging situations	Knowledge and skills needed for the management of a challenging situation
You can discuss that with other teachers; now it seems like this, and we could act like that	Discuss with other teachers about how to act	Collegial support	Type of support needed for the management of challenging situations
I think that there should be a mental health nurse on our staff	A mental health nurse working at the school	Support from psychiatric professionals in schools	Suggestions for improvement
